# Some Clinical Features of Mediastinal Disease

**Published:** 1903-06-06

**Authors:** 


### SOME CLINICAL FEATURES OF MEDIASTINAL DISEASE.

The possible affections of the mediastinum are many; new growths, either innocent or malignant, may arise from any of its structures; the mediastinal glands may be the seat of chronic or acute inflammation; abscesses may form as the result of infection; the connective tissues of the mediastinum may be infected by one of the granulomata, giving rise to indurative mediastinitis; parasitic cysts sometimes form in the mediastinum; and aneurysms, both true and false, occur in this region with considerable frequency.

Certain general symptoms are found to occur as a result of one or other of these lesions of the mediastinum. The aspect, attitude, and state of nutrition of the patient are all points worthy of careful consideration. Mediastinal diseases often cause some degree of cyanosis, and with this there may be enlargement of the superficial veins of the thorax. In these enlarged veins the direction of the blood flow is often abnormal, establishing a circulation between the veins of the head and neck and those of the trunk. Orthopnœa, in which the patient has to live, eat, and sleep in a sitting posture, is another symptom connected with diseases of the mediastinum. The state of nutrition of the patient often throws important light on the nature of a tumour of the mediastinum, though progressive emaciation is of more importance than the actual extent of malnutrition.

But together with these general symptoms must be taken the associated conditions, such as lesions elsewhere. These, when infective in nature, may give rise to specific secondary lesions of the mediastinum. The age, sex, and life history of the patient must all be factors in determining the probability of any particular lesion. The effects of treatment on the existing lesion are often sufficient to demonstrate its nature and character. Particularly is this the case in syphilitic lesions when treated with iodide of potassium, though the beneficial results of potassium iodide in the treatment of actinomycosis must not be forgotten.

The local symptoms of mediastinal disease may be due either to the primary lesion or an associated condition. For example, dyspnœa may be caused by the pressure of a tumour on the trachea, or the displacement of the heart by an aneurysm of the aorta; but dyspnœa is quite as easily caused by the pleural effusion that may arise as a secondary complication in either of these conditions.

Pressure on important veins is evidenced by the consequent obstruction to the circulation of the blood. Venous engorgement thus caused is an important symptom, indicating the probable position of the lesion. Dysphagia and regurgitation of food both give valuable information on the same point.

The physical signs of disease within the mediastinum may not be clear or well defined. Certain areas of impaired resonance may be detected. The physical signs of the heart and lungs will also indicate the nature and extent of the lesion. The cardiac impulse is usually displaced, and the auscultatory signs correspondingly altered. Certain adventitious sounds can often be heard, owing to the obstruction to the blood flow. The air entry into one or both lungs will be impaired, and possibly one lung, or portion of lung, will be in a condition of collapse.

Further important and additional information can be obtained by the use of the Roentgen rays. The extent of the tumour, and its connection or otherwise with the heart and great blood-vessels, can be demonstrated by this means. Indeed, the examination of any case of mediastinal disease is hardly complete without an $x$-ray photograph, or demonstration with the screen.

The mediastinal affections more commonly met with are mediastinal tumour, or aneurysm of the aorta. Aneurysm is characterised by a fixed pain of a boring character. The pain is continuous, and has acute radiations. It is increased by exertion, and this is well marked in the most fixed aneurysms. Among the more important signs of aneurysm are abnormal pulsation, visible or palpable ; a tumour over which a thrill or diastolic shock may be felt; certain pressure signs, unequal pupils, hoarseness, etc. ; abnormalities of circulation, unequal radial or carotid pulses; loud low-pitched aortic second sound ; and a systolic murmur heard best over the tumour. In this connection it may be remembered that the aneurysm of the first part of the arch causes physical signs, that of the second part is accompanied by symptoms, while that of the third part is more particularly associated with severe pain in the middle of the back.

Tumours invading the mediastinum give rise to pain, but it is usually less acute than with aneurysm, and also is not increased by exertion to the same extent. The dyspnœa caused by a mediastinal aneurysm is frequently paroxysmal, but in the case of new growths the dyspnœa is more continuous. Venous obstruction is, however, more marked with tumours of the mediastinum than with aneurysm. On the other hand a persistent hard brassy cough is more closely connected with aneurysms than mediastinal tumours. The cough is probably due to irritative lesions of the vagus nerve. Hæmoptysis may be a symptom alike in cases of aneurysm, or cases of tumour, when it is due to passive congestion of the lung, or more rarely direct hæmorrhage from the aneurysm of the aorta.

Indurative mediastinitis is one of the rarer lesions of the mediastinum. Generally associated with adherent pericardium and pleuritic adhesions, it is but a continuation of a general fibro-plastic process. It may, however, occur as a single local lesion, and in this case it is frequently syphilitic in origin. The symptoms of indurative mediastinitis closely resemble those of mediastinal tumour, but with certain differences. In the history and course of the disease characteristic points may be noted. It occurs in young adults under 30 years of age. The onset is insidious and protracted.

It is usually a sequel to the pleurisy and pericarditis of early life. Pleural and peritoneal effusion are often present, and venous obstruction is especially conspicuous. The jugular vein is frequently obstructed, causing venous engorgement of the head and neck. The liability to cardiac lesions is greater in these cases, and dyspnœa is often very marked, exertion being impossible.

In cases of abscess of the mediastinum, pain is perhaps the most prominent symptom, though the cough is frequently most trying. Venous obstruction is less marked, though there are other local signs, *e.g.*, tenderness, swelling, and œdema. General signs will also indicate the nature of the lesion, such as pyrexia and rigors. Associated conditions, as possible causes of the abscess, must be carefully looked for, *e.g.*, ulceration of the œsophagus, bronchiectasis, pneumonia, pleurisy, tuberculosis, pyæmia, or direct injury may be the indirect but exciting cause of mediastinal abscess.

In infants and young children the thymus may become adenomatous, reaching a large size. Cases have been recorded where an enlarged thymus has given rise to the symptoms and signs of mediastinal disease. There is obstructive dyspnœa, but the dulness is more extensive than in other forms of mediastinal disease.

The several points mentioned above must be taken together and weighed against one another before the diagnosis of any case of mediastinal disease can become even probable.

